# The Optimal Lipid Chain Length of a Membrane-Permeabilizing Lipopeptide Results From the Balance of Membrane Partitioning and Local Damage

**DOI:** 10.3389/fmicb.2021.669709

**Published:** 2021-09-14

**Authors:** Jessica Steigenberger, Yentl Verleysen, Niels Geudens, José C. Martins, Heiko Heerklotz

**Affiliations:** ^1^Department of Pharmaceutical Technology and Biopharmacy, University of Freiburg, Freiburg, Germany; ^2^NMR and Structure Analysis Research Group, Department of Organic and Macromolecular Chemistry, Ghent University, Ghent, Belgium; ^3^Leslie Dan Faculty of Pharmacy, University of Toronto, Toronto, ON, Canada; ^4^Signaling Research Centers BIOSS and CIBSS, University of Freiburg, Freiburg, Germany

**Keywords:** cyclic lipodepsipeptides, membrane-permeabilizing, structure-function relationship, *Pseudomonas*, pseudodesmin A, liposome leakage, lipid chain length, equi-activity analysis

## Abstract

Pseudodesmin A (PSD) is a cyclic lipodepsipeptide produced by *Pseudomonas* that kills certain bacteria at MIC_1/2_ in the single micromolar range, probably by permeabilizing their cellular membranes. Synthetic PSD variants, where the native decanoic (C10) acyl chain is varied in length from C4 to C8 and C12 to C14 carbons, were described to be not or less active against a panel of gram-positive strains, as compared to native PSD-C10. Here, we test the membrane-permeabilizing activity of PSD-C4 through PSD-C14 in terms of calcein release from liposomes, which is characterized in detail by the fluorescence-lifetime based leakage assay. Antagonistic concentrations and their chain length dependence agree well for liposome leakage and antimicrobial activity. The optimal chain length is governed by a balance between membrane partitioning (favoring longer chains) and the local perturbation or “damage” inflicted by a membrane-bound molecule (weakening for longer chains). Local perturbation, in turn, may involve at least two modes of action. Asymmetry stress between outer and inner leaflet builds up as the lipopeptides enter the outer leaflet and when it reaches a system-specific stability threshold, it causes a transient membrane failure that allows for the flip of some molecules from the outer to the inner leaflet. This cracking-in may be accompanied by transient, incomplete leakage from the aqueous cores of the liposomes observed, typically, for some seconds or less. The mismatch of the lipopeptide with the lipid leaflet geometry, expressed for example in terms of a spontaneous curvature, has two effects. First, it affects the threshold for transient leakage as described. Second, it controls the rate of equilibrium leakage proceeding as the lipopeptide has reached sufficient local concentrations in both leaflets to form quasi-toroidal defects or pores. Both modes of action, transient and equilibrium leakage, synergize for intermediate chain lengths such as the native, i.e., for PSD-C10. These mechanisms may also account for the reported chain-length dependent specificities of antibiotic action against the target bacteria.

## Introduction

Naturally occurring lipopeptides and other membrane-active compounds may have great potential for a more individual, selective design of antimicrobials, insecticides, plant protection agents, and drugs and are considered a “gold mine” (Cochrane and Vederas, 2016) and a highly promising approach to resolve growing resistances and loss of biodiversity, even though their modes of action are yet still partially enigmatic (Balleza et al., 2019; Wimley and Hristova, 2019). Besides classic antimicrobial peptides, which are often produced via ribosomal synthesis (Datta et al., 2014; Kormilets et al., 2019), the diverse collective of membrane-active biomolecules also includes cyclic lipopeptides (CLiPs) (Raaijmakers et al., 2006; Kleijn and Martin, 2018) synthesized non-ribosomally by bacteria such as *Bacillus* (Ongena and Jacques, 2008) or *Pseudomonas* (Geudens et al., 2017; Geudens and Martins, 2018). They consist of a (partially) cyclic oligopeptide group attached to a lipid chain and are amphipathic. These CLiPs may show plant beneficial, antimicrobial, antiviral, antitumoral, or antifungal activity, presumably by perturbing cellular membranes (Raaijmakers et al., 2010; Schneider et al., 2014; Bonnichsen et al., 2015; Komin et al., 2017; Li et al., 2017; Guha et al., 2019).

The impact of the lipidic tail length on biological activity has already been noticed in classic studies based on the separation of individual species from natural mixtures comprising a variety of chain lengths. Ongena and co-workers (Henry et al., 2011) have demonstrated that surfactins, a family of CLiPs from *Bacillus*, were able to trigger an immune reaction based on hydrogen peroxide release from tobacco plant cells if they included a sufficiently long lipidic chain with 14 or 15 carbons (denoted C14 and C15) while the C12 and C13 variants showed almost no activity. Furthermore, it has been reported that the anti-*Candida* activity of bacillomycin-D-like lipopeptides, also produced by *Bacillus*, increased with chain length from the C14 to the C15 and further to the C16 tail variant (Tabbene et al., 2011). Similar results have been found by Eshita et al. (1995) for bacillomycin-L homologs (C14 to C16) while investigating their anti-fungal activity. However, since these studies employed naturally occurring variants, they were limited both in scale and in scope.

Recently, De Vleeschouwer et al. (2020) studied the effect of the lipidic chain length of the CLiP pseudodesmin A (PSD) from *Pseudomonas* on antimicrobial activity using variants obtained through total synthesis. The native pseudodesmin A with its 10-carbon chain (denoted PSD-C10 here) turned out to be active against all six tested strains from *Enterococcus*, *Staphylococcus*, and *Streptococcus* genera with minimal inhibitory concentrations that inhibit 50% bacterial growth (MIC_1/2_) of 1–4 μg/ml. Synthetic, shorter chain analogs turned out less active (PSD-C8) or inactive (PSD-C6 and PSD-C4). The longer chain analogs PSD-C12 and PSD-C14 retained full activity against four of the six strains. The two genera *Enterococcus faecium* and *Staphylococcus aureus* were inhibited exclusively by the native PSD-C10.

**FIGURE 1:**
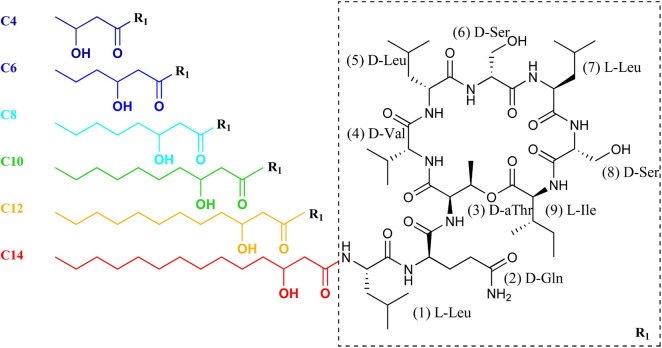
Molecular structure of the pseudodesmin A analogs PSD-C4 to PSD-C14. The color code of each 3-hydroxy fatty acid chain indicates the respective analog (C4–C14) throughout the study.

Here, the access to these synthetic PSD lipid tail variants (Figure 1) is exploited for a biophysical investigation of the impact of lipid tail length on membrane permeabilization. The first objective of our study is to test whether the occurrence of a minimal or optimal chain length for antimicrobial activity of PSD analogs is paralleled by an optimal chain length for the permeabilization of liposomal model membranes. The second objective is to provide detailed, mechanistic, and quantitative insight on which parameters govern such an optimal CLiP chain length. To test the hypothesis that these chain-length effects may be due to rather unspecific bilayer interactions, we selected POPC liposomes as a generic membrane model examining only this level of interactions. This approach deliberately eliminates other important, potential determinants of lipid selectivity (Fiedler et al., 2015) that may arise from specific lipids contained in the outer and inner leaflet of the plasma membranes of gram-positive bacteria. This aims at assigning chain length effects on activity and selectivity to their underlying molecular interactions; it does not attempt modeling the bacterial membrane as good as possible.

The strategy of our study is to separate the contributions to dose-response curves of membrane permeabilization arising from (i) membrane partitioning and (ii) the extent of local damage caused by membrane-inserted molecules. In this context, “local damage” stands for any local structure or event induced by a membrane-inserted PSD that gives rise to membrane leakage. It does not distinguish between specific modes of action such as the potential formation of pore-forming PSD assemblies, (which have only been observed in chloroform) (Sinnaeve et al., 2009a), the formation of double-stranded PSD-filaments (Crowet et al., 2019) or the rather unspecific induction of lipid bilayer stress that results in leakage.

This partitioning versus local damage strategy is inspired by the finding that other hydrophobic size optima of membrane-active compounds are a result of a balance between membrane insertion (favoring long chains) on the one hand and higher local activity within the membrane (favoring shorter chains) on the other hand. This principle applies, at least to some extent, to membrane permeabilization (de la Maza et al., 1998) and solubilization (Heerklotz, 2008) by detergents. Also the famous Meyer-Overton rule (Meyer, 1899; Overton, 1901), which describes the increase in activity of general anesthetics with increasing lipophilicity, is limited by a loss of local activity of too lipophilic compounds (Cantor, 2001; Ingólfsson and Andersen, 2011). Finally, a common window of optimal hydrophobicity was also found for the vast majority of drugs that have to diffuse across membrane barriers to reach the blood stream (Lipinski et al., 2001; Lipinski, 2004), in this case resulting from the need to dissolve sufficiently in both, hydrophilic and lipophilic, environments.

In this work, membrane leakage is characterized using a fluorescence-lifetime based leakage assay (Patel et al., 2009) which combines the self-quenching of entrapped calcein (Blumenthal et al., 1977; Allen and Cleland, 1980) enabling simultaneous quantification and characterization of free and entrapped dye utilizing time-correlated single-photon counting (TCSPC) without neglecting the potentially increasing fluorescence intensity from dye remaining entrapped. An equi-activity analysis is performed to separate effects of partitioning from those of local damage (Encinas and Lissi, 1982; Heerklotz et al., 1994; Paternostre et al., 1995; de la Maza and Parra, 1997; Heerklotz and Seelig, 2007; Fan et al., 2014). To this end, liposome leakage is measured as a function of both, lipid and PSD concentrations. As a result, a clearer picture emerges for the role of the lipidic chain length in the expression of antimicrobial activity.

## Materials and Methods

### Materials

Lipoid GmbH (Ludwigshafen, Germany) kindly provided the phospholipid 1-palmitoyl-2-oleoyl-sn-glycero-3-phosphocholine (POPC). Calcein and ethylenediaminetetraacetic acid (EDTA) were purchased from Sigma-Aldrich (St. Louis, MO, United States). Tris(hydroxymethyl)aminomethane (Tris), chloroform, dimethyl sulfoxide (DMSO), NaCl, NaOH, and HCl were purchased from Carl Roth GmbH (Karlsruhe, Germany).

The cyclic lipopeptide pseudodesmin A (PSD-C10) was isolated as previously described (Sinnaeve et al., 2009b). Its synthetic analogs PSD-C4, PSD-C6, PSD-C8, PSD-C12, PSD-C14, solubilized in DMSO, were synthesized as described (De Vleeschouwer et al., 2014, 2020) and quantified by NMR using the digital ERETIC method based on PULCON (Wider and Dreier, 2006). All solutions were prepared using ultrapure water prepared by the arium^®^ pro system (Sartorius AG, Göttingen, Germany).

### Liposome Preparation

Large unilamellar POPC vesicles encapsulating the fluorescence dye calcein (calcein-LUVs) were prepared by thin lipid film hydration method. First, dry lipids were dissolved in chloroform and pipetted in desired amounts into 1.5 mL HPLC vials using a positive displacement pipette (Eppendorf AG, Hamburg, Germany). Chloroform was removed by a RVC 2–18 CDplus vacuum centrifugator at 36°C (Martin Christ GmbH, Osterode am Harz, Germany) and thin lipid films were dried under high vacuum overnight. Dried lipid films were covered with inert argon gas and vial lids were sealed with parafilm. Lipid films were either stored at −20°C or used for liposome preparation immediately.

Subsequently, lipid films were hydrated with calcein buffer (70 mM calcein, 10 mM Tris, 0.5 mM EDTA, pH 7.4) at room temperature, followed by four freeze-thaw cycles using dry ice and a water bath at 50°C, and extruded with a LiposoFast hand extruder (Avestin Inc., Ottawa, ON, Canada) through a 200 nm (30×) and a 100 nm polycarbonate membrane (51×). Z-Average hydrodynamic diameter (115–125 nm) and polydispersity index (<0.1) were checked by dynamic light scattering (DLS) using a Zetasizer Nano ZS (Malvern Panalytical Ltd., Worcestershire, United Kingdom). LUVs were loaded on a PD-10 desalting column (GE Healthcare, Little Chalfont, United Kingdom) to remove free calcein and to exchange the external calcein buffer by isotonic standard buffer (10 mM Tris, 110 mM NaCl, 0.5 mM EDTA, pH 7.4). The final phospholipid concentration was analyzed via colorimetric determination of inorganic phosphate according to Bartlett (1959) and liposomes were stored at room temperature to avoid phase transition.

### Leakage Kinetics Experiments

First, a dispersion of calcein-LUVs was filled into disposable fluorescence cuvettes (Sarstedt AG & Co., KG, Nümbrecht, Germany). Then, an aliquot of the respective PSD solution in DMSO was slowly added and gently mixed with the liposome dispersion to start the incubation time. The final lipid concentration in these samples was 30 μM and the final DMSO content was ≤3 v%. The samples were incubated on a rotary shaker (400 rpm) at 25°C and protected from light. TCSPC measurements as described below were carried out practically immediately after mixing and after incubation for 10 min, 30 min, 1 h, 2 h, and 24 h. Samples without any PSD, containing only Tris buffer and calcein-LUVs, were always included as controls.

Here, the use of stock solutions in DMSO to enhance dissolution and mixing of PSD with the liposome suspension follows the procedure utilized by, e.g., Coraiola et al. (2006) or Geudens et al. (2017). Small aliquots of these stock solutions are injected into liposome dispersions to start the incubation and, potentially, the leakage process (“peptide-into-lipid” mixing). The final DMSO concentration upon incubation was ≤3 v%. Reference experiments at various DMSO contents ranging from 1 to 20 v% indicated that at up to 5 v%, DMSO does not affect CLiP-induced liposome leakage.

### Dose-Response Curves at Variable Lipid Concentrations

Experiments at various lipid concentrations were performed following a top-up protocol as described (Heerklotz and Seelig, 2007; Patel et al., 2011; Fiedler and Heerklotz, 2015). This procedure allows to study the effect of different lipid concentrations during the incubation time of the liposomes with the PSDs on the one hand but conserves the lipid and the calcein concentrations upon TCSPC measurement on the other hand. This approach also keeps inner filter effects and turbidity constant for all measurements, which is particularly important for steady-state experiments. TCSPC is unaffected by general intensity changes but we nevertheless followed this optimal protocol, which also reduces the total amount of calcein-LUVs needed. The dilution upon top-up must be considered to induce the release of bound peptide from the liposomes, thus slowing down or quenching further leakage. Hence, diluted samples do not permit recording kinetic data by repeated measurements of the same sample.

According to this protocol, specific amounts of PSD were incubated with calcein-LUVs all containing 42 nmol of lipid, either in 210 μL (200 μM lipid), in 420 μL (100 μM lipid), in 700 μL (60 μM lipid), or in 1,400 μL (30 μM lipid), respectively. These samples were prepared directly in disposable cuvettes (Sarstedt AG & Co., KG, Nümbrecht, Germany) and shaken. Prior to the measurement, all samples were topped up with standard buffer to 1.4 mL, thus diluting the lipid to 30 μM and the PSD in proportion.

In addition to the top-up series, a set of samples was measured after incubating the PSDs with 15 μM lipid in 1.4 mL without top-up, i.e., with half the lipid and calcein concentrations compared to the 30 μM series, and with 1 μM lipid in 1.4 mL (for PSD-C10 and PSD-C12) without top-up.

### Time-Correlated Single-Photon Counting (TCSPC)

PSD-induced calcein leakage from LUVs was measured as described (Patel et al., 2009) by TCSPC in a FluoTime 100 spectrometer (PicoQuant, Berlin, Germany). Excitation was at 467 nm (pulse width 20 ps) using an LDH-P-C-470 laser diode (PicoQuant, Berlin, Germany) operated by a PDL 800-D laser driver with a 20 MHz repetition rate. Pile-up was kept below 1 count per 100 excitation pulses (sync) by a suitable attenuator. Fluorescence was accumulated at ≥530 nm (OG530 longpass filter) by a PMA 175-N detector for 30 s, giving rise to about 10^5^ peak counts, and recorded with a resolution of 25 ps per bin. For each series of runs, the instrument response function (IRF) was recorded using a Ludox HS-40 scattering standard.

Using the FluoFit software (PicoQuant), the TCSPC data were fitted by reconvolution of the IRF with a biexponential decay of the amplitude, B, as a function of time after the trigger signal of each excitation pulse, t:


(1) $$B\left(t\right)=B_{E}\cdot e^{-\frac{t}{\tau_{E}}}+B_{F}\cdot e^{-\frac{t}{\tau_{F}}}$$

Here, τ_*F*_ ≈ 4 ns stands for the fluorescence lifetime of dilute calcein, i.e., the dye outside the liposomes. After complete leakage, the concentration of the free dye is about 5 μM. Dynamic self-quenching reduces the lifetime of liposome-entrapped dye, τ_*E*_, to 0.4 ns at the original dye concentration of 70 mM. Partial release of dye from the liposomes and the resulting gradual dilution cause intermediate values of 0.4 ns < τ_*E*_ < 4 ns and, hence, alter the contribution of the entrapped dye to the fluorescence intensity. The same would apply in case of potential interactions of some calcein with the inner liposome surface as described for carboxyfluorescein (Chen and Knutson, 1988). However, the preexponential factors, B_*F*_ and B_*E*_, obtained from the fit scale to a good approximation with the total numbers of free and entrapped dye molecules, respectively, thus yielding the fraction of leaked dye, L, as:


(2) $$L=\frac{B_{F}-B_{F0}}{B_{F}-B_{F0}+Q_{Stat}\cdot B_{E}}$$

Here, B_*F0*_ represents free calcein present without leakage (due to imperfect removal of free calcein by size exclusion chromatography) and,


(3) $$Q_{Stat}=\frac{B_{F}-B_{F0}}{B_{E0}-B_{E}}\approx 1.2\pm 0.2$$

corrects for a small contribution of static quenching of entrapped calcein (see Patel et al., 2009 for details).

## Results

### Test of Assay and Error Assessment

Preliminary experiments showed that PSDs became inactivated for membrane permeabilization when attempted to be dissolved in buffer with ≤3 v% of DMSO. What turned out possible was a peptide-into-lipid mixing assay injecting very small aliquots of PSD solutions in DMSO into a liposome dispersion. As the DMSO gets diluted, the PSD can bind to liposomes and, hence, its precipitation is avoided. A general drawback of this approach compared to the “lipid-into-peptide” protocol (Wieprecht et al., 1999; Heerklotz and Seelig, 2007; Patel et al., 2011) is that it does not exclude locally and transiently higher PSD concentrations interacting with liposomes during the mixing process, typically on a sub-second timescale. Such heterogeneity effects may affect leakage data if the redistribution of the peptide to equilibrate between the liposomes is not faster than leakage.

**FIGURE 2:**
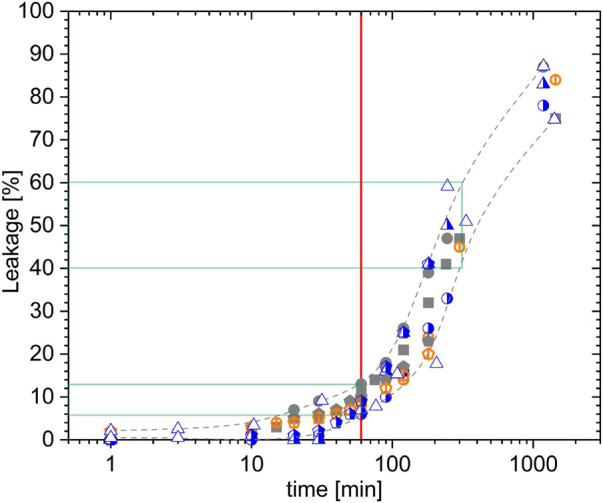
Error analysis for different “peptide-into-lipid” mixing protocols. Leakage kinetics data for 25 μM PSD-C14 acting on liposomes of 30 μM POPC (=lipid) as a function of incubation time. Gray solid symbols represent mixing sequences with 30 μM lipid + PSD-C14, open orange symbols with vertical bar denote the sequence of 15 μM lipid + PSD-C14 + 15 μM lipid (so far, all liposomes calcein loaded), and half-blue symbols represent the sequence of 15 μM lipid without calcein + PSD-C14 + 15 μM lipid calcein loaded. Different symbol types with the same color/style stand for individual series of experiments. Grid lines point to the 1 h standard incubation time (red) and typical L errors after 1 and 5 h (green).

First, we tested experimental error ranges that include not only variations observed by simple repetitions but also possible shifts due to mixing heterogeneity issues. To this end, we recorded leakage kinetics in samples incubating 25 μM PSD-C14, the compound with the lowest solubility in our study, with liposomes of 30 μM POPC (Figure 2). Gray symbols represent samples with calcein-loaded liposomes (30 μM lipid) to which the PSD was added (standard “peptide-into-lipid” protocol), either in a gentler fashion or with the sample on the rotation mixer upon pipetting the PSD-C14. Half-blue symbols were obtained by starting with a 15 μM POPC liposomal dispersion containing no calcein, i.e., not contributing to the leakage readout. Then, the PSD-C14 was added and right after, another aliquot of liposomes, this time calcein-loaded ones, adding another 15 μM lipid. The overall contents after mixing are the same as before, 30 μM lipid exposed to 25 μM PSD-C14, but the PSD that has bound to the “empty” liposomes upon injection can leak the calcein-loaded liposomes only after redistributing between the liposomes. Finally, orange symbols with a vertical bar refer to the sequence of additions starting with 15 μM calcein-loaded liposomes and adding, first, 25 μM PSD-C14 and, second, another 15 μM calcein-loaded liposomes.

**FIGURE 3:**
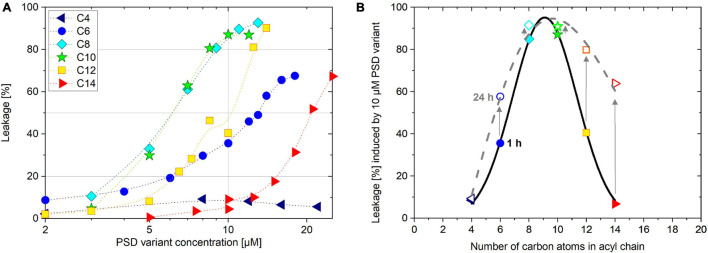
Induced leakage of 30 μM calcein-LUV after 1 h incubation time as a function of PSD concentration **(A)**. Leakage of 30 μM calcein-LUV induced by 10 μM PSD as a function of PSD acyl chain length after 1 h (filled symbols) and 24 h (open symbols) incubation time **(B)**.

Inspection of the results shows likely significant deviations up to about 30 min incubation for the “empty-PSD-calcein” protocol (half-blue symbols), indicating that redistribution of PSD-C14 proceeds on the timescale of minutes. After 1 h, all data seem to scatter within the same range, *L* = (9 ± 2 SD) % and after 5h, *L* = (50 ± 10)%. Note that the crucial error range of this assay is not so much that of L but rather that of the active concentration at which significant, e.g., 50%, leakage occurs, c_*PSD*_^50^. For the curves shown in Figure 3A, an error of ±10% in L would convert into ±10% in c_*PSD*_^50^.

Slow redistribution of a PSD can be seen from two perspectives. On the one hand, it could be considered an experimental artifact if the measured extents and kinetics of leakage are affected by the speed of the peptide to actually reach each liposome equally. On the other hand, compounds with poor solubility in the range of their active concentration will also suffer from reduced bioavailability and, hence, reduced or delayed activity *in vivo*.

The other crucial error in this study is that of the intrinsically active concentration of bound PSD. This is obtained from the statistics of the fit of the equi-activity lines as explained in section “Equi-Activity Analysis” below.

### Typical Dose-Response Curves

Dose response curves represent a defined response (here: calcein leakage after a given incubation time) as a function of the active agent (here: PSD concentration) at a set target concentration (here: lipid concentration). In line with literature standards (Wimley and Hristova, 2019) and considering practicability, we decided to use an incubation time of 60 min and a set lipid concentration of 30 μM calcein-LUV to measure typical dose-response curves for all PSD analogs (Figure 3A).

The most leakage-active PSD analogs according to this criterion were, interestingly, the natural compound with its 10-carbon chain, PSD-C10, and its C8 analog. They show sigmoidal and rather steep dose-response curves centered at c_*PSD*_ (*L* = 50%) ≡ c_*PSD*_^50^ ≈ 6 μM, increasing from 20 to 80% leakage over a factor of about 2 in concentration (the typical step in an MIC assay). Analogs with longer acyl chains appear less active, the associated curves shifting to the right (see c_*PSD*_^50^ in Table 1) while keeping their slope roughly unchanged. Shortening the chain rendered the compounds less active as well. Furthermore, PSD-C6-induced leakage was less cooperative as inferred from the less steep dose-response curve compared to the longer chain analogs. The PSD-C4 analog carrying the shortest lipid chain shows almost no membrane-permeabilization activity anymore up to a concentration of 22 μM.

**TABLE 1:** Effective PSD concentrations to induce 20, 50, and 80% calcein leakage of 30 μM calcein-LUVs after 1 and 24 h incubation time.

**PSD**	**1 h incubation**			**24 h incubation**		
	**c_*PSD*_^50^**	**c_*PSD*_^20^**	**c_*PSD*_^80^**	**c_*PSD*_^50^**	**c_*PSD*_^20^**	**c_*PSD*_^80^**
	**All in μM**					
C4	>20	>20	>20	>20	>20	>20
C6	13	6	>18	8	4	16
C8	6	4	9	4	2	6
C10	6	4	9	4	3	6
C12	10	6	13	6	4	10
C14	21	15	>25	8	5	13

*The data represent individual measurements, for a general analysis of errors see section “Test of Assay and Error Assessment.”*

The chain length dependence of the leakage-inducing activity is moreover illustrated by the amount of calcein leakage of 30 μM calcein-LUVs induced by 1 h preincubation with 10 μM of the PSD analogs (Figure 3B, filled symbols). Plotting leakage, L, as a function of the PSD acyl chain length yields a bell-shaped activity curve. After 24 h incubation, the activity maximum remains at PSD-C10 as already seen after 1 h. However, the longer-chain compounds PSD-C12 and PSD-C14 “catch up,” inducing more calcein leakage with time due to their slower yet ongoing leakage (Figure 3B, open symbols; see also section on kinetics below).

### Leakage Kinetics

**FIGURE 4:**
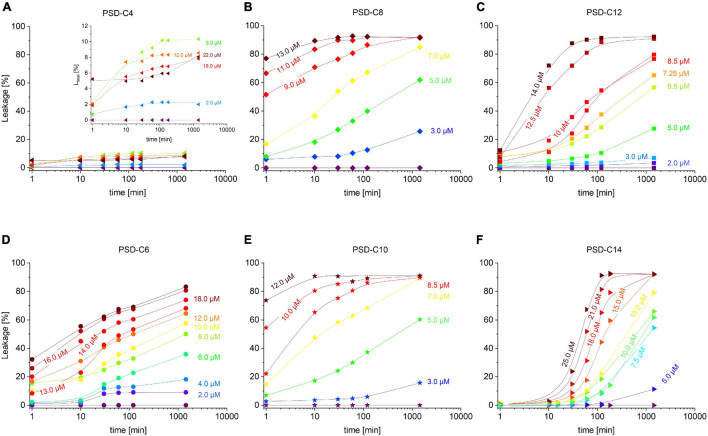
Cumulative leakage kinetics over 24 h of calcein-LUVs (=30 μM) induced by PSD-C4 **(A)**, PSD-C6 **(D)**, PSD-C8 **(B)**, PSD-C10 **(E)**, PSD-C12 **(C)**, and PSD-C14 **(F)**. The amount of total leakage (=released calcein) in % is shown as a function of time on a logarithmic scale. Investigated time points are right upon addition of the active compound and after 10, 30, 60, 120, (180 for PSD-C4 and PSD-C14), and 1440 min incubation time. Lines are to guide the eye, whereas the rainbow color code changes from purple (=0 μM PSD addition) to dark red with increasing concentrations of the respective active PSD variant, as labeled.

Dose-response curves refer to a snapshot of leakage after a defined incubation time, in our example 60 min. This raises the question of how fast an antimicrobial agent should kill its target bacteria to be active *in vivo*. Different incubation times may compromise the comparison between different assays of activity. Furthermore, the progress of leakage as a function of incubation time can also provide additional information about the mode of action of the compound (Hovakeemian et al., 2015; Stulz et al., 2019). Therefore, we have recorded leakage kinetics over several hours at a constant *c*_*L*_ = 30 μM for all PSDs studied here (Figure 4) to reflect the occurrence and effectiveness of leakage events over time. The curves reveal different types of leakage kinetics which have been classified by Wimley and Hristova (2019) as transient, equilibrium, and hybrid leakage.

Transient leakage results from a non-equilibrium state of the membrane that equilibrates, at least partially, upon leakage so that leakage stops after a transient, limited efflux of dye. A typical cause of transient leakage is asymmetry stress between a peptide-containing outer and a peptide-free inner leaflet of the bilayer; for a detailed discussion, see below. By contrast, “equilibrium leakage” may occur within a relaxed, relatively equilibrated membrane that does not change its principal properties associated with the dye release. Individual defects or pores may close after some time, but others will appear with the same likelihood. Ideally, equilibrium leakage proceeds with first order kinetics until practically all dye is released. Hybrid leakage is a combination of the two.

The kinetics in Figure 4 imply that most PSDs show hybrid leakage yet with proportions changing markedly with chain length. Transient leakage accounts for the limited levels of leakage by PSD-C4 reached within approximately 10 min. It then becomes faster and more pronounced with increasing chain length as evident from the increasing “starting” leakage, L (*t* ≈ 1 min), for PSD-C6 and PSD-C8. For PSD-C10, there is no marked further increase compared to PSD-C8. For PSD-C12 and PSD-C14, this fast leakage becomes very weak and eventually vanishes.

Substantial equilibrium leakage is seen for PSD-C6 through -C14, with slower rates for long-chain analogs, PSD-C12 and -C14, compared to PSD-C8 and -C10. As discussed in the section “Materials and Methods,” it cannot be ascertained that the kinetics of PSD-C14 leakage, particularly on the minute timescale, are unaffected by a slow redistribution of the molecule after heterogeneously binding to liposomes upon mixing. The finding of a significant lag time between mixing and the beginning of leakage and the exact rates may be caused by this phenomenon.

As mentioned, this is not just a technical issue as slow redistribution and limited bioavailability due to low solubility must be considered to affect biological function, too. General modes of action related to such kinetics as well as potential implications for antimicrobial activity will be discussed below.

### Equi-Activity Analysis

At first glance, it appears counterintuitive that a continuous change in the chain length of PSD analogs causes a bell-shaped activity profile as illustrated in Figure 3B. A likely explanation of this behavior is that membrane leakage requires a balance of (i) membrane partitioning, which is a prerequisite for any action within the membrane and should increase with increasing chain length, and (ii) the extent of local damage induced by a membrane-bound CLiP contributing to leakage.

To test this hypothesis, we have separated the contributions of partitioning and local damage to the dose-response curves by an equi-activity analysis (Heerklotz, 2008; Patel et al., 2009, 2011). This analysis assumes that a certain membrane change (here: leakage) is unequivocally related to the content of an active compound within the membrane, specified here by the mole ratio of membrane-bound PSD *c_*PSD*_^*b*^* to lipid *c*_*L*_, *R*_*b*_:


(4) $$R_{b}=\frac{c_{PSD}^{b}}{c_{L}}$$

**FIGURE 5:**
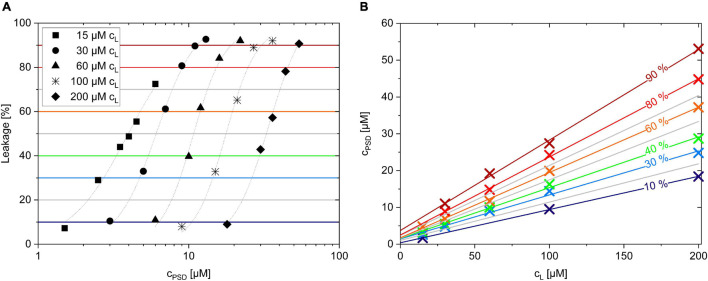
Leakage after 1 h incubation of PSD-C8 with calcein-LUVs with lipid concentrations *c*_*L*_ ranging from 15 to 200 μM **(A)**. Plotting c_*PSD*_ needed to trigger a certain calcein leakage (%) for a given lipid concentration, c_*L*_, creates the equi-activity plot of PSD-C8 **(B)**.

In other words, whenever a certain leakage, *L*, is measured, the membrane contains a corresponding ratio *R*_*b*_ of PSD per lipid. Practically, a certain degree of leakage can be obtained by different combinations of total PSD and lipid concentrations, *c*_*PSD*_ and *c*_*L*_. Figure 5A shows leakage curves for five different lipid concentrations (*c*_*L*_ = 15–200 μM). Each curve reaches a certain *L* value, for example, *L* = 40% (green horizontal line), at a specific *c*_*PSD*_. The (interpolated) crossing points of e.g., the green horizontal line for *L* = 40% with the leakage curves are then plotted as green multiplication signs in Figure 5B.

The fact that the crossing points describe a straight line to a very good approximation is a direct consequence of the assumption that all these points, sharing, e.g., *L* = 40%, also share a common *R*_*b*_, which shall be referred to as *R*_*b*_^40^. Namely, the total PSD concentration *c*_*PSD*_ is the sum of the concentrations of bound and aqueous PSD, *c_*PSD*_^*b*^* and *c_*PSD*_^*aq*^*, and the former can be replaced by *R_*b*_⋅c_*L*_*. Using eq. (4), we then obtain the linear relationship:


(5) $$c_{PSD}^{40}=R_{b}^{40}\cdot c_{L}+c_{PSD}^{aq,40}$$

The green line representing *c_*PSD*_^40^* as a function of *c*_*L*_ in Figure 5B with:


(6) $$c_{PSD}^{40}=0.14\cdot c_{L}+1.6\mu M$$

implies that ∼14 bound molecules of PSD-C8 per 100 POPC molecules (*R_*b*_^40^*, slope) induce 40% calcein leakage after 1 h, and that this membrane is in equilibrium with a free PSD-C8 concentration of 1.6 μM (y-intercept). The same procedure was carried out for all other horizontal lines in Figure 5A yielding the linear fits in Figure 5B and, in turn, series of corresponding L, R_*b*_, and c_*PSD*_^*aq*^ values for each PSD as listed in Table 2.

**TABLE 2:** R_*b*_^50^, c_*PSD*_^*aq,50*^ [μM], and K [μM^–1^] values for PSD analogs.

**PSD**	**R_*b*_^50^**	**c_*PSD*_^*aq,50*^ [μM]**	**K [μM^–1^]**
C6	0.17(1*h*)0.043	7.03.0	0.0300.0067
C8	0.16(1*h*)0.0034	1.60.36	0.0890.0035
C10	0.18(1*h*)0.0029	1.30.32	0.140.007
C12	0.27(1*h*)0.014	1.40.87	0.280.020
C14	0.55(3*h*)0.042	Set to 0	n.d.

**FIGURE 6:**
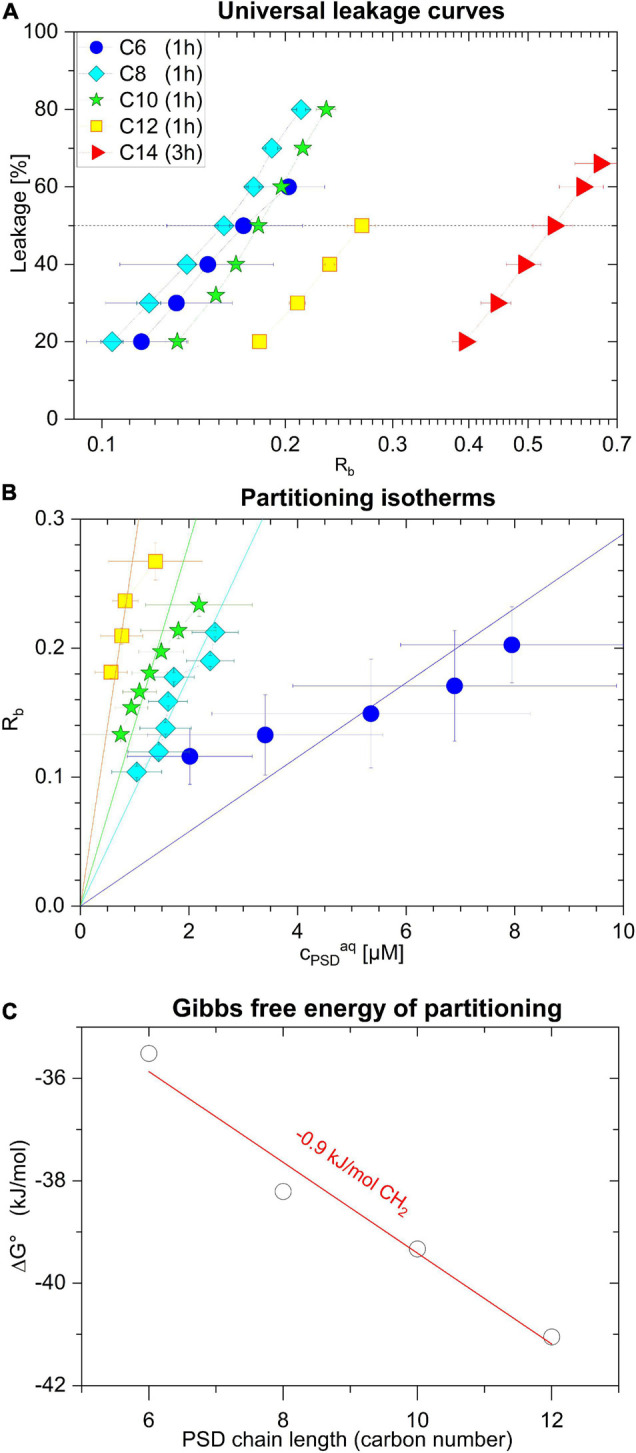
Leakage of calcein-LUVs after 1 h incubation time induced by PSD-C6 (blue circle), PSD-C8 (cyan diamond), PSD-C10 (green star), and PSD-C12 (yellow square), and after 3 h incubation time induced by PSD-C14 (red triangle), as a function of the mole ratio within the lipid membrane, R_*b*_ **(A)**. R_*b*_ as a function of free PSD (c_*PSD*_^*aq*^) describing membrane partitioning for C6, C8, C10, and C12 analogs **(B)**. The standard Gibbs free enthalpy of partitioning, ΔG°, as a function of the PSD chain length along with a linear fit suggesting a methylene group contribution of –0.9 kJ/mol **(C)**. Error bars indicate standard errors of the equi-activity fit as illustrated in Figure 5.

*R_*b*_^50^ and c_*PSD*_^*aq,50*^ [μM] derived by equi-activity analysis after 1 h (C6-C12) or 3 h (C14) incubation time. K [μM^–1^] values derived by linear regression of respective R_*b*_ and c_*PSD*_^*aq*^ [μM] data and shown by the slope and Y-Intercept of the linear fits in Figure 5B. The very weak calcein leakage induced by PSD-C4 at relevant concentrations did not warrant an equi-activity analysis. For PSD-C14, the intercepts in the equi-activity fit, (c_*PSD*_(c_*L*_); c_*PSD*_^*aq*^), were too small (within error of zero) to be quantified so that K could not be calculated. Error ranges represent standard errors of the equi-activity fit (as illustrated in Figure 5) for R_*b*_^50^ and c_*PSD*_^*aq,50*^ and of the fit of the partitioning isotherms shown in Figure 6B. Additional errors may apply as discussed in section “Test of Assay and Error Assessment.”*

It should be noted that a complete equi-activity analysis reaches its limits for compounds with very high membrane affinity that yield y-intercepts of the equi-activity plot (displaying c_*PSD*_ as a function of c_*L*_ as shown in Figure 5B), i.e., values of c_*PSD*_^*aq*^, that are within the error of the origin (Fan et al., 2014; Fiedler and Heerklotz, 2015). This problem occurred for PSD-C14. The resolution of the low-c_*L*_-range is limited by the signal-to-noise ratio of the experiments. Due to this, a free linear fit of the PSD-C14 data to calculate R_*b*_ and c_*PSD*_^*aq*^ was not possible. Hence, the origin of the linear fits for the PSD-C14 data were set to 0 for further analysis. Then, the analysis still provides a good value for R_*b*_ (which, in this case, approaches the total mole ratio in the sample) but does not warrant quantifying c_*PSD*_^*aq*^ and, in turn, the partition coefficient (see below).

## Discussion

### Activity as a Balance Between Membrane Insertion, Local Membrane Damage, and Bioavailability

Performing the equi-activity analyses supports our hypothesis that the bell-shaped leakage-activity curve (Figure 3B), indicating an optimal PSD chain length of 8–10 carbons for POPC membrane permeabilization, is a consequence of the balance between partitioning (favoring long chains, see partitioning isotherms in Figure 6B) and induced local membrane damage (favoring short chains, see universal leakage curves in Figure 6A). Furthermore, very low solubility of longer-chain compounds was discussed to limit bioavailability and slow down kinetics of membrane insertion and leakage.

Strong local damage imposed by a membrane-bound PSD is indicated, e.g., by a low *R_*b*_^50^*. In fact, *R_*b*_^50^* decreases with decreasing chain length from C14 to C8 (Figure 6A and Table 2); large uncertainties for PSD-C6 due to imperfect leakage curve resolution obscure a possible trend as chain length is further reduced. It should be noted that the construction of the universal leakage curve eliminates the effect of the absolute lipid concentrations used in the experiments, but these leakage curves still change as a function of incubation time. That means, slow leakage contributes little to the local damage by long-chain PSDs after 60 min, but these PSDs may “catch up” to some extent with increasing incubation times.

The partitioning isotherms (Figure 6B) show that the affinity of the PSDs to insert into the membrane increases monotonically with increasing chain length. The slope of an error-weighted linear fit through the origin yields the average, apparent mole-ratio partition coefficient, K, as described in Lichtenberg (1985), de la Maza et al. (1998), Heerklotz (2008):


(7) $$K=\frac{R_{b}}{c_{PSD}aq}$$

The resulting values for K are included in Table 2 and the resulting standard Gibbs free enthalpy of partitioning, ΔG°, is plotted as a function of chain length in Figure 6C. Note that the slope reveals a standard Gibbs free energy increment of ΔΔG° per methylene of −0.9 kJ/mol, which is considerably smaller than those concluded from hydrocarbon solubility (−3.7 kJ/mol) (Gill and Wadso, 1976), micelle formation (−3.1 kJ/mol) (Heerklotz and Epand, 2001) and membrane partitioning of detergents (3.4 kJ/mol) (Heerklotz, 2008). This is reminiscent of a reduced increment found for micelle formation of short-chain diacyl phosphatidylcholines (−2.5 kJ/mol) (Tanford, 1980; Heerklotz and Epand, 2001) which was explained by chain-chain contacts causing reduced water exposure in solution. Analogously, we may explain this finding for PSDs by the chain folding back to hydrophobic regions of the peptide moiety in solution, as stated, e.g., for the CLiP Tolaasin I by Jo (2011).

### The Effect of PSD Chain Length on the Mode of Membrane-Permeabilizing Action

As mentioned already, membrane leakage by PSDs is based on at least two distinct modes of action, with contributions that vary depending on the PSD chain length.

**FIGURE 7:**
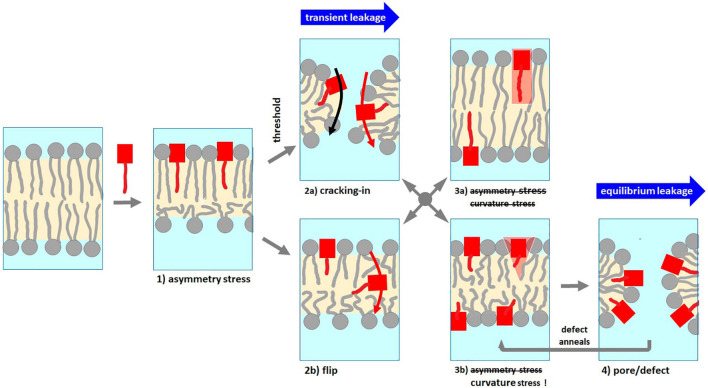
Schematic representation of the expected membrane phenomena and their related modes of action to induce leakage by PSDs. The data suggest PSD-C4 to act by asymmetry stress and to cause transient leakage accompanying its cracking-in (trace 1-2a-3a). Due to its weak membrane partitioning, PSD-C4 fails to reach the local content required to induce pores via curvature stress. PSD-C6 through PSD-12 induce transient leakage translocating PSD into the inner leaflet that gives rise to curvature strain, hence, promotes subsequent equilibrium leakage (trace 1-2a-3b-4-3b-4 etc.). PSD-C14 shows no fast, transient leakage but, after slow flip of some of it to the inner leaflet, equilibrium leakage (trace 1-2b-3b-4-3b-4 etc.). See text for further explanation.

While leakage data do not provide direct information about the local PSD and lipid arrangements of membrane defects or pores, they may provide indispensable, complementary information on modes of action. For example, the finding of transient, limited leakage can be considered a strong indication for a mode of action that is eliminating its own driving force, such as asymmetry stress. Accordingly, the PSD inserts between the lipids in the outer leaflet of the membrane bilayer and, thus, expands this outer leaflet (cartoon 1 in Figure 7). If the bilayer cannot bend, the areas of both leaflets have to match and hence, the inner leaflet gets stretched well beyond its optimal area. This creates a mechanical area-asymmetry stress (Heerklotz, 2001; Heerklotz et al., 2004; Esteban-Martín et al., 2009) [for reviews: see Heerklotz (2008); Wimley and Hristova (2019)], that opposes further PSD uptake (Heerklotz, 2001; Heerklotz and Seelig, 2001; Fan et al., 2016). This asymmetry stress can, ultimately, be relaxed either by (i) a flip of individual molecules across the intact membrane (cartoon 2b in Figure 7) or by (ii) a transient local failure of the membrane that permits a “cracking-in” of peptide and lipid (Fan et al., 2014, 2016) (cartoon 2a in Figure 7) and is often accompanied by leakage between the aqueous compartments at both sides of the membrane as well. As the asymmetry stress is relaxed at least partially, the membrane anneals and remaining entrapped dye can no longer be released via this asymmetry stress driven mechanism (cartoon 3a in Figure 7; Pokorny and Almeida, 2004). The respective cartoons in Figure 7 are inspired by snapshots of an MD simulation of this mechanism by Esteban-Martín et al. (2009) and Marrink et al. (2009).

In addition to this transient, typically fast but limited leakage, PSDs show a second, often slower but unlimited leakage process which can tentatively be assigned to a local arrangement of PSDs and/or lipids that induces a defect or pore. Examples for such structures are pore-forming peptide oligomers, which have in fact been proposed also for PSD (Sinnaeve et al., 2009a, 2012; Crowet et al., 2019), and quasi-toroidal defects of the lipid bilayer (cartoon 4 in Figure 7) with its high local interfacial curvature stabilized by curvature-active peptides (Esteban-Martín et al., 2009; Marrink et al., 2009). The latter phenomenon would be in line with a weakening of the local damage with increasing chain length because longer chains give rise to less positive spontaneous curvature. However, it is also conceivable that the formation of pore forming peptide assemblies favors shorter chains.

Taking all these findings together provides convincing, possible explanations for the chain-length optimum for the antibacterial activity of PSDs at intermediate chain lengths such as decyl:

(i) Membrane partitioning increases with increasing chain length. Of PSDs with shorter chains, a major fraction typically remains in solution and these molecules are not active in the membrane.

(ii) Local damage tends to decrease with increasing chain length. This is expected concerning curvature activity but may also be related to a lower tendency to self-assemble to form pores.

(iii) Bioavailability may become compromised for long-chain PSDs on shorter timescales. Low solubility may slow down PSD transport to and into the target membrane.

### Antibacterial Activity and Selectivity-Suggestions From Liposome Leakage

**FIGURE 8:**
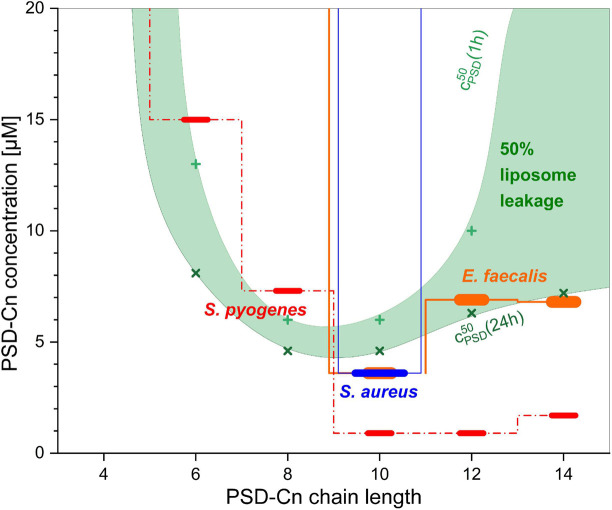
Comparison of the minimal inhibitory concentrations which inhibited bacterial growth by at least 50% (MIC_1/2_) of PSD analogs against selected bacteria (step functions with horizontal bars) as published by De Vleeschouwer et al., 2020, and the concentrations needed to induce 50% leakage from 30 μM calcein-LUVs after 1 h (+, upper border of green area) and after 24 h (×, lower border). All characteristic concentrations are shown as a function of the chain length of the PSD analogs.

The concentrations of the PSDs needed for liposome leakage agree astonishingly well with the minimal inhibitory concentrations which inhibit bacterial growth by at least 50% (MIC_1/2_) against a panel of Gram-positive bacteria reported by De Vleeschouwer et al. (2020), as illustrated by Figure 8. The concentration range used for antibacterial testing extends from 0.016 to 32 μg/mL in twelve dilution steps which converts to ≈ 30 μM as the highest tested concentration (exact concentrations range from 27.1 μM (PSD-C14) to 32.5 μM (PSD-C4) due to minor differences in molar mass). Both *S. aureus* (blue bar in Figure 8) and *E. faecium* (not shown in Figure 8) are inhibited at concentrations below ≈ 30 μM only by the natural PSD-C10, with all other chain length variants showing MIC_1/2_ values above this limit. Moreover, the C10-chain also represents a threshold chain length for *Enterococcus faecalis* (orange bars in Figure 8), *Clostridium perfringens*, and *Streptococcus pneumonia* (not shown in Figure 8) as PSDs with shorter chain lengths display MIC_1/2_ values equal to or above the 30 μM limit against these three organisms. For PSD analogs with longer chains, the activity against these three bacterial strains is comparable to the observed activity for the natural PSD-C10. Finally, *Streptococcus pyogenes* (red dashed bars in Figure 8) is sensitive to a broader range of PSDs, with PSD-C10 and PSD-C12 being the most active species.

The concentrations needed to induce 50% dye leakage from 30 μM POPC calcein-LUVs after 1 h (light green crosses in Figure 8) to 24 h (dark green multiplication signs in Figure 8) are used to define the boundaries of the green area in Figure 8. For lower lipid concentrations, the boundaries would shift down, approaching for c_*L*_→0 the values of c_*PSD*_^*aq,50*^ which are given in Table 2. Based on the hypotheses discussed in the previous section, one could assign the upper boundary of the green range to rather fast, primarily asymmetry-stress driven mode of action and the lower boundary to also include equilibrium defect formation.

These boundaries not only agree with the general concentration range of the MIC_1/2_ but also, to a large extent, with the observed chain length dependency and with their minimum MIC_1/2_ for the C10 chain. Furthermore, a previous study of liposome leakage induced by native PSD on a specific model for bacterial membranes yielded fairly similar results as well. Leakage of liposomes of phosphatidylglycerol, phosphatidylethanolamine and cardiolipin (7:2:1 mole ratio, total 25 μM) progressed between 2–8 μM PSD (c_*PSD*_^50^ = 4 μM) (Geudens et al., 2017) as compared to 3–10 μM (c_*PSD*_^50^ = 6 μM) for 30 μM of POPC obtained here.

In spite of these agreements, the results reported here should be discussed with utmost caution. As discussed above, the simple lipid bilayer model used in this work is not meant to represent at all potentially important features of inner membranes of Gram-positive bacteria. Instead, it demonstrates that the chain length dependence obtained for several MICs can be generally explained already on the lowest level of lipid bilayer complexity. The good quantitative agreement of c_*PSD*_^50^ with that of more elaborate model membranes and with MIC_1/2_ values may be fortuitous given expected influences of the specific lipid composition of the bacterial membrane (Sohlenkamp and Geiger, 2016), non-lipid effects of bacterial targets, the open question which extent of leakage corresponds to a deadly damage, et cetera. Nevertheless, the generic phenomena suggested by our data to govern leakage allow for hypotheses regarding modes of action and selectivity criteria to inspire related, microbiological tests.

Equilibrium leakage requires a threshold content of PSD in the inner leaflet and often proceeds at a slow rate. It could be opposed by efflux pumps or other mechanisms to eliminate the PSD from the inner leaflet on the time scale of hours. One may speculate that *S. aureus* and *E. faecium* are able to resist slow, equilibrium leakage whereas *E. faecalis* with its MIC_1/2_ values rather following the lower, slow-leakage boundary of the green area, is not.

Fast, transient leakage by asymmetry stress would be avoided by other membrane components that can quickly redistribute between the leaflets hence balancing the outside insertion of PSDs by accumulating these other compounds in the inner membrane leaflet. Alternatively, resistance could arise from a “blow-off valve” allowing for the controlled breakthrough of lipids to the inner leaflet above a threshold stress, maybe similar to mechanosensitive channels limiting symmetric membrane stretching in hypoosmotic environments (Peyronnet et al., 2014). Finally, an overall higher mechanical stability of the membrane may help tolerating asymmetry stress. Maybe *S. pyogenes* is less able to utilize such mechanisms than, e.g., *S. aureus* which requires a combination of maximal asymmetry stress and reasonably fast equilibrium leakage, as triggered presumably by the natural PSD-C10, to be killed.

The maximal activity of PSD-C10 to leak membranes was explained in the previous section on the basis of maximal transient leakage, fastest equilibrium leakage and a synergism between the two, along with sufficient solubility. This combination of modes of action may render susceptible microorganisms more prone to membrane failure while also making it more difficult for microorganisms to develop resistance.

## Conclusion

The availability of a homologous series of lipid chain analogs of a cyclic lipopeptide, provided by total synthesis, opens a new avenue for the detailed, quantitative study of structure-function relationships.

The native pseudodesmin A with its lipid chain length of C10 is also the most active chain-length variant of the series PSD-C4 – PSD-C14 to permeabilize zwitterionic POPC lipid membranes.

The bell-shaped activity curve as a function of chain length of the PSD analogs is explained by the balance between membrane partitioning (increasing with increasing chain length), the local damage caused by a membrane-inserted PSD (decreasing for longer chains) and bioavailability and kinetic effects of solubility (potentially limiting function for long chains).

Intermediate chain length variants act by a, potentially synergistic, combination of two modes of action: transient leakage to relax bilayer asymmetry stress and equilibrium leakage due to local defects or pores induced by membrane destabilizing arrangements of PSD or of lipids along with PSD.

Although here we only consider generic bilayer effects devoid of particularly characteristic components of bacterial membranes, the active concentrations for liposome leakage by the different PSD analogs agree astonishingly well with inhibitory concentrations against certain bacteria.

## Data Availability Statement

The datasets generated for this study are available on request to the corresponding author.

## Author Contributions

JS performed the experiments, analyzed the data, and wrote the first draft of the manuscript. NG isolated PSD (C10) from natural sources. YV prepared the samples of the synthetic variants. All authors designed the research, discussed the results, and finalized the manuscript.

## Conflict of Interest

The authors declare that the research was conducted in the absence of any commercial or financial relationships that could be construed as a potential conflict of interest.

## Publisher’s Note

All claims expressed in this article are solely those of the authors and do not necessarily represent those of their affiliated organizations, or those of the publisher, the editors and the reviewers. Any product that may be evaluated in this article, or claim that may be made by its manufacturer, is not guaranteed or endorsed by the publisher.
